# 4-Meth­oxy-*N*-(4-meth­oxy-2-nitro­phen­yl)benzamide

**DOI:** 10.1107/S1600536812037701

**Published:** 2012-09-29

**Authors:** Muhammad Arshad, Sammer Yousuf, Sumayya Saeed, Fatima Z. Basha

**Affiliations:** a H.E.J. Research Institute of Chemistry, International Center for Chemical and Biological Sciences, University of Karachi, Karachi 75270, Pakistan; b Department of Chemistry, University of Karachi, Karachi 75270, Pakistan

## Abstract

In the title compound, C_15_H_14_N_2_O_5_, the central amide C—C(=O)—N—C unit forms dihedral angles of 28.17 (13) and 26.47 (13)° with the two benzene rings, whereas the two benzene rings are almost coplanar, making a dihedral angle of 4.52 (13)°. The two meth­oxy and the nitro substituents are almost coplanar with their attached benzene rings, with C—O—C—C torsion angles of −1.3 (4) and −4.6 (4)°, and an O—N—C—C torsion angle of 17.1 (3)°. In the crystal, mol­ecules are linked *via* C—H⋯O and N—H⋯O inter­actions, forming a tape running along the *b* axis.

## Related literature

For the crystal structures of related benzamide compounds, see: Sripet *et al.* (2012 ▶); Saeed *et al.* (2008 ▶); Saeed & Flörke (2009 ▶).
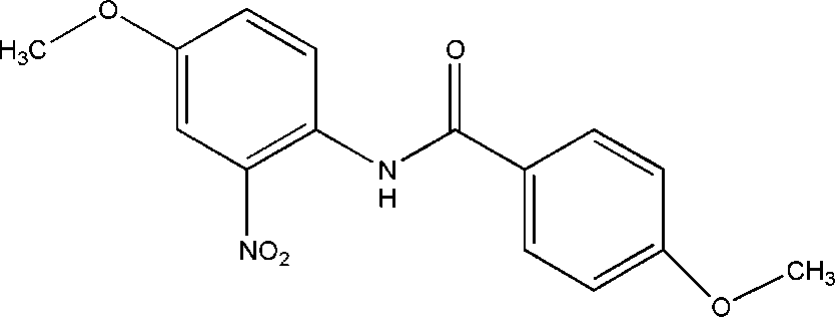


## Experimental

### 

#### Crystal data

C_15_H_14_N_2_O_5_
*M* *_r_* = 302.28
Monoclinic, 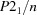

*a* = 9.7206 (12) Å
*b* = 4.9885 (6) Å
*c* = 28.725 (4) Å
β = 95.628 (2)°
*V* = 1386.2 (3) Å^3^
*Z* = 4
Mo *K*α radiation
μ = 0.11 mm^−1^
*T* = 273 K
0.30 × 0.12 × 0.07 mm

#### Data collection

Bruker SMART APEX CCD area-detector diffractometer
Absorption correction: multi-scan (*SADABS*; Bruker, 2000 ▶) *T*_min_ = 0.968, *T*_max_ = 0.992
7491 measured reflections
2552 independent reflections
1638 reflections with *I* > 2σ(*I*)
*R*_int_ = 0.049

#### Refinement

*R*[*F* ^2^ > 2σ(*F* ^2^)] = 0.064
*wR*(*F* ^2^) = 0.155
*S* = 1.00
2552 reflections
205 parameters
H atoms treated by a mixture of independent and constrained refinement
Δρ_max_ = 0.24 e Å^−3^
Δρ_min_ = −0.19 e Å^−3^

### 

Data collection: *SMART* (Bruker, 2000 ▶); cell refinement: *SAINT* (Bruker, 2000 ▶); data reduction: *SAINT*; program(s) used to solve structure: *SHELXS97* (Sheldrick, 2008 ▶); program(s) used to refine structure: *SHELXL97* (Sheldrick, 2008 ▶); molecular graphics: *SHELXTL* (Sheldrick, 2008 ▶); software used to prepare material for publication: *SHELXTL*, *PARST* (Nardelli, 1995 ▶) and *PLATON* (Spek, 2009 ▶).

## Supplementary Material

Crystal structure: contains datablock(s) global, I. DOI: 10.1107/S1600536812037701/is5189sup1.cif

Structure factors: contains datablock(s) I. DOI: 10.1107/S1600536812037701/is5189Isup2.hkl

Supplementary material file. DOI: 10.1107/S1600536812037701/is5189Isup3.cml

Additional supplementary materials:  crystallographic information; 3D view; checkCIF report

## Figures and Tables

**Table 1:** Hydrogen-bond geometry (Å, °)

*D*—H⋯*A*	*D*—H	H⋯*A*	*D*⋯*A*	*D*—H⋯*A*
N1—H1*A*⋯O2^i^	0.80 (3)	2.34 (3)	3.027 (3)	145 (3)
C10—H10*A*⋯O5^ii^	0.93	2.45	3.364 (3)	168

Symmetry codes: (i) 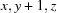; (ii) 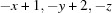.

**Fig. 1.**
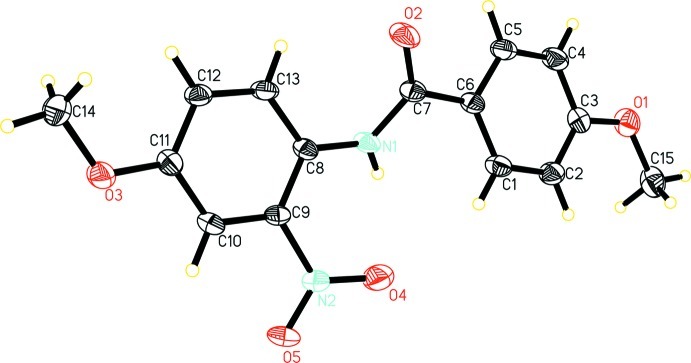
The molecular structure of the title compound with displacement ellipsoids drawn at 30% probability level.

**Fig. 2.**
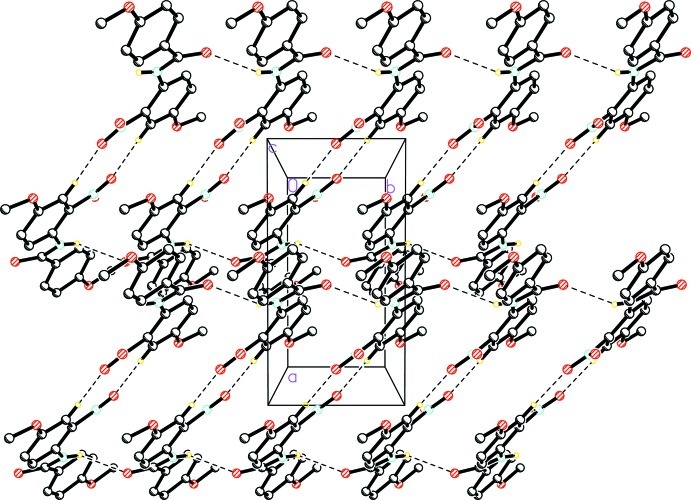
The crystal packing of the title compound. Hydrogen atoms are omitted for clearity. Dashed lines indicate the C—H···O and N—H···O hydrogen bonds.

## supplementary crystallographic information

### Comment

The formation of amide functionality is a fundamental reaction and of great interest in organic chemistry. Due to a number of application in industrial and pharmaceutical areas as well as an important intermediate in synthetic chemistry, it remains a great challenge for the chemists to develop an efficient method for the synthesis of amides. The title compound was obtained during our attempt to synthesize libraries of benzamide derivatives under different conditions.

The molecule of title compound is not planner. The central amide unit (C6/C7/O2/N1/C8) forms dihedral angles of 28.17 (13) and 26.47 (13)°, respectively, with benzene C1–C6 and C8–C13 rings. The dihedral angle between the two C1–C6 and C8–C13 rings is 4.52 (13)°. The two methoxy and the nitro susbtituents lie nearly in plane of the corresponding aromatic rings with torsion angles C14—O3—C11—C12 of -4.6 (4)°, C15—O1—C3—C2 of -1.3 (4)°, O5—N2—C9—C10 of 17.1 (4)° and O4—N2—C9—C10 of -161.3 (3)°. The bond lengths and angles are similar to those found in the related benzamide derivatives (Sripet *et al.*, 2012; Saeed *et al.*, 2008; Saeed & Flörke, 2009). In the crystal, molecules are linked *via* intermolecular C—H···O and N—H···O (symmetry codes as in Table 2) interactions to form an infinite tape structure running along the *b* axis (Fig. 2).

### Experimental

The title compound was synthesized by using the following procedure. To the stirring solution of 4-methoxy-2-nitroaniline (2.97 mmol) in 6.5 ml dichloromethane, *p*-methoxybenzoyl chloride (8.12 mmol) and triethylamine (0.5 ml) were added carefully at room temperature. The progress of reaction was checked by TLC and was completed in 12 h. Then the reaction mixture was diluted with water (25 ml) and acidified with 1.0 *M* HCl (50 ml). The organic compound was extracted with ethyl acetate (2×25 mL) and washed with brine (50 ml). The organic layer was dried (anhyd. MgSO_4_), filtered and concentrated on rotavapor. The crude mixture was purified by silica gel column chromatography by using ethyl acetate and hexane to get title compound with 77% yield. After column chromatography, the pure compound was left overnight. The crystals obtained were found suitable for single-crystal X-ray diffraction studies. All chemicals were purchased from Sigma-Aldrich and Alfa Aesar.

### Refinement

The H atom on the nitrogen was located in a difference Fourier maps and refined freely [N—H = 0.80 (3) Å]. Other H atoms were positioned geometrically with 0.93 or 0.96 Å and constrained to ride on their parent atoms with *U*_iso_(H) = 1.2*U*_eq_(C) or 1.5*U*_eq_(methyl C). A rotating group model was applied to the methyl groups.

### Crystal data

**Table:**

C_15_H_14_N_2_O_5_	*F*(000) = 632
*M**_r_* = 302.28	*D*_x_ = 1.448 Mg m^−^^3^
Monoclinic, *P*2_1_/*n*	Mo *K*α radiation, λ = 0.71073 Å
Hall symbol: -p 2yn	Cell parameters from 1456 reflections
*a* = 9.7206 (12) Å	θ = 2.3–28.0°
*b* = 4.9885 (6) Å	µ = 0.11 mm^−^^1^
*c* = 28.725 (4) Å	*T* = 273 K
β = 95.628 (2)°	Plate, colorles
*V* = 1386.2 (3) Å^3^	0.30 × 0.12 × 0.07 mm
*Z* = 4	

### Data collection

**Table:**

Bruker SMART APEX CCD area-detector diffractometer	2552 independent reflections
Radiation source: fine-focus sealed tube	1638 reflections with *I* > 2σ(*I*)
Graphite monochromator	*R*_int_ = 0.049
ω scan	θ_max_ = 25.5°, θ_min_ = 2.2°
Absorption correction: multi-scan (*SADABS*; Bruker, 2000)	*h* = −9→11
*T*_min_ = 0.968, *T*_max_ = 0.992	*k* = −6→6
7491 measured reflections	*l* = −34→34

### Refinement

**Table:**

Refinement on *F*^2^	Primary atom site location: structure-invariant direct methods
Least-squares matrix: full	Secondary atom site location: difference Fourier map
*R*[*F*^2^ > 2σ(*F*^2^)] = 0.064	Hydrogen site location: inferred from neighbouring sites
*wR*(*F*^2^) = 0.155	H atoms treated by a mixture of independent and constrained refinement
*S* = 1.00	*w* = 1/[σ^2^(*F*_o_^2^) + (0.0824*P*)^2^] where *P* = (*F*_o_^2^ + 2*F*_c_^2^)/3
2552 reflections	(Δ/σ)_max_ < 0.001
205 parameters	Δρ_max_ = 0.24 e Å^−^^3^
0 restraints	Δρ_min_ = −0.19 e Å^−^^3^

### Special details

**Table:**

Geometry. All e.s.d.'s (except the e.s.d. in the dihedral angle between two l.s. planes) are estimated using the full covariance matrix. The cell e.s.d.'s are taken into account individually in the estimation of e.s.d.'s in distances, angles and torsion angles; correlations between e.s.d.'s in cell parameters are only used when they are defined by crystal symmetry. An approximate (isotropic) treatment of cell e.s.d.'s is used for estimating e.s.d.'s involving l.s. planes.
Refinement. Refinement of *F*^2^ against ALL reflections. The weighted *R*-factor *wR* and goodness of fit *S* are based on *F*^2^, conventional *R*-factors *R* are based on *F*, with *F* set to zero for negative *F*^2^. The threshold expression of *F*^2^ > σ(*F*^2^) is used only for calculating *R*-factors(gt) *etc*. and is not relevant to the choice of reflections for refinement. *R*-factors based on *F*^2^ are statistically about twice as large as those based on *F*, and *R*- factors based on ALL data will be even larger.

### Fractional atomic coordinates and isotropic or equivalent isotropic displacement parameters (Å^2^)

**Table:**

	*x*	*y*	*z*	*U*_iso_*/*U*_eq_	
O1	−0.0633 (2)	0.6081 (4)	0.30064 (6)	0.0569 (6)	
O2	0.0516 (2)	0.0858 (3)	0.10748 (7)	0.0621 (7)	
O3	0.3257 (2)	0.4247 (4)	−0.08001 (6)	0.0560 (6)	
O4	0.3544 (2)	0.8107 (5)	0.11160 (7)	0.0759 (8)	
O5	0.4308 (2)	1.0026 (4)	0.05315 (7)	0.0629 (7)	
N1	0.1306 (2)	0.5046 (4)	0.09575 (8)	0.0413 (6)	
N2	0.3622 (2)	0.8309 (4)	0.06979 (8)	0.0415 (6)	
C1	0.1170 (3)	0.6004 (5)	0.19444 (9)	0.0431 (7)	
H1B	0.1889	0.6872	0.1815	0.052*	
C2	0.0863 (3)	0.6723 (5)	0.23865 (9)	0.0465 (7)	
H2A	0.1383	0.8033	0.2554	0.056*	
C3	−0.0216 (3)	0.5495 (5)	0.25793 (9)	0.0415 (7)	
C4	−0.0970 (3)	0.3527 (6)	0.23291 (9)	0.0508 (8)	
H4A	−0.1709	0.2708	0.2456	0.061*	
C5	−0.0633 (3)	0.2782 (5)	0.18956 (9)	0.0488 (8)	
H5A	−0.1130	0.1415	0.1736	0.059*	
C6	0.0434 (3)	0.4016 (4)	0.16870 (8)	0.0361 (6)	
C7	0.0754 (3)	0.3157 (4)	0.12181 (8)	0.0368 (6)	
C8	0.1817 (3)	0.4747 (4)	0.05195 (8)	0.0340 (6)	
C9	0.2883 (3)	0.6356 (4)	0.03780 (8)	0.0329 (6)	
C10	0.3324 (3)	0.6180 (5)	−0.00614 (8)	0.0381 (7)	
H10A	0.4017	0.7313	−0.0146	0.046*	
C11	0.2736 (3)	0.4317 (5)	−0.03782 (8)	0.0386 (7)	
C12	0.1694 (3)	0.2690 (5)	−0.02481 (9)	0.0404 (7)	
H12A	0.1297	0.1416	−0.0456	0.048*	
C13	0.1236 (3)	0.2943 (5)	0.01899 (9)	0.0413 (7)	
H13A	0.0511	0.1864	0.0266	0.050*	
C14	0.2750 (3)	0.2204 (6)	−0.11201 (9)	0.0567 (8)	
H14A	0.3227	0.2301	−0.1397	0.085*	
H14B	0.1778	0.2458	−0.1202	0.085*	
H14C	0.2905	0.0479	−0.0976	0.085*	
C15	0.0108 (4)	0.8074 (6)	0.32821 (9)	0.0627 (9)	
H15A	−0.0276	0.8246	0.3576	0.094*	
H15B	0.0037	0.9758	0.3120	0.094*	
H15C	0.1062	0.7563	0.3336	0.094*	
H1A	0.138 (3)	0.648 (6)	0.1083 (9)	0.059 (9)*	

### Atomic displacement parameters (Å^2^)

**Table:**

	*U*^11^	*U*^22^	*U*^33^	*U*^12^	*U*^13^	*U*^23^
O1	0.0677 (16)	0.0606 (12)	0.0453 (11)	−0.0009 (11)	0.0196 (10)	−0.0054 (10)
O2	0.1059 (19)	0.0283 (9)	0.0559 (12)	−0.0171 (10)	0.0273 (11)	−0.0071 (9)
O3	0.0702 (16)	0.0543 (11)	0.0473 (11)	−0.0215 (10)	0.0250 (10)	−0.0120 (9)
O4	0.085 (2)	0.0929 (16)	0.0514 (13)	−0.0457 (14)	0.0168 (11)	−0.0209 (12)
O5	0.0706 (17)	0.0463 (11)	0.0716 (14)	−0.0343 (11)	0.0066 (11)	0.0025 (10)
N1	0.0570 (18)	0.0242 (11)	0.0452 (13)	−0.0096 (10)	0.0184 (11)	−0.0070 (10)
N2	0.0403 (16)	0.0338 (11)	0.0504 (14)	−0.0078 (10)	0.0051 (11)	−0.0041 (11)
C1	0.0422 (19)	0.0399 (14)	0.0492 (16)	−0.0091 (13)	0.0147 (13)	−0.0034 (13)
C2	0.056 (2)	0.0392 (14)	0.0449 (15)	−0.0088 (13)	0.0094 (14)	−0.0082 (13)
C3	0.046 (2)	0.0384 (14)	0.0413 (15)	0.0061 (13)	0.0110 (13)	0.0037 (12)
C4	0.049 (2)	0.0525 (16)	0.0533 (17)	−0.0147 (14)	0.0177 (14)	0.0016 (14)
C5	0.057 (2)	0.0408 (14)	0.0493 (16)	−0.0179 (14)	0.0113 (14)	−0.0033 (13)
C6	0.0405 (18)	0.0255 (11)	0.0432 (14)	−0.0013 (11)	0.0083 (12)	0.0005 (11)
C7	0.0398 (18)	0.0256 (12)	0.0457 (14)	−0.0004 (11)	0.0085 (12)	−0.0031 (11)
C8	0.0384 (18)	0.0248 (11)	0.0400 (14)	−0.0019 (11)	0.0110 (12)	0.0002 (10)
C9	0.0321 (17)	0.0226 (11)	0.0438 (14)	−0.0023 (10)	0.0026 (12)	−0.0019 (10)
C10	0.0377 (18)	0.0307 (12)	0.0477 (15)	−0.0057 (12)	0.0126 (12)	0.0034 (11)
C11	0.0444 (19)	0.0325 (12)	0.0405 (14)	0.0013 (12)	0.0122 (12)	−0.0002 (11)
C12	0.0469 (19)	0.0299 (12)	0.0454 (15)	−0.0085 (12)	0.0100 (13)	−0.0080 (11)
C13	0.0434 (19)	0.0334 (12)	0.0488 (15)	−0.0147 (12)	0.0127 (13)	−0.0068 (12)
C14	0.068 (2)	0.0585 (18)	0.0457 (16)	−0.0103 (16)	0.0147 (15)	−0.0122 (14)
C15	0.097 (3)	0.0487 (16)	0.0445 (16)	0.0080 (17)	0.0159 (17)	−0.0035 (14)

### Geometric parameters (Å, º)

**Table:**

O1—C3	1.361 (3)	C5—C6	1.391 (4)
O1—C15	1.422 (3)	C5—H5A	0.9300
O2—C7	1.232 (3)	C6—C7	1.475 (3)
O3—C11	1.359 (3)	C8—C13	1.386 (3)
O3—C14	1.427 (3)	C8—C9	1.402 (3)
O4—N2	1.215 (3)	C9—C10	1.375 (3)
O5—N2	1.212 (3)	C10—C11	1.384 (3)
N1—C7	1.348 (3)	C10—H10A	0.9300
N1—C8	1.405 (3)	C11—C12	1.378 (4)
N1—H1A	0.80 (3)	C12—C13	1.381 (3)
N2—C9	1.476 (3)	C12—H12A	0.9300
C1—C2	1.380 (3)	C13—H13A	0.9300
C1—C6	1.392 (3)	C14—H14A	0.9600
C1—H1B	0.9300	C14—H14B	0.9600
C2—C3	1.377 (4)	C14—H14C	0.9600
C2—H2A	0.9300	C15—H15A	0.9600
C3—C4	1.384 (4)	C15—H15B	0.9600
C4—C5	1.370 (4)	C15—H15C	0.9600
C4—H4A	0.9300		
			
C3—O1—C15	118.2 (2)	C13—C8—N1	121.6 (2)
C11—O3—C14	117.2 (2)	C9—C8—N1	122.3 (2)
C7—N1—C8	128.2 (2)	C10—C9—C8	122.4 (2)
C7—N1—H1A	113 (2)	C10—C9—N2	116.0 (2)
C8—N1—H1A	118 (2)	C8—C9—N2	121.7 (2)
O5—N2—O4	122.6 (2)	C9—C10—C11	120.1 (2)
O5—N2—C9	118.2 (2)	C9—C10—H10A	120.0
O4—N2—C9	119.2 (2)	C11—C10—H10A	120.0
C2—C1—C6	121.8 (3)	O3—C11—C12	125.1 (2)
C2—C1—H1B	119.1	O3—C11—C10	116.0 (2)
C6—C1—H1B	119.1	C12—C11—C10	118.9 (2)
C3—C2—C1	119.8 (2)	C11—C12—C13	120.3 (2)
C3—C2—H2A	120.1	C11—C12—H12A	119.9
C1—C2—H2A	120.1	C13—C12—H12A	119.9
O1—C3—C2	125.0 (2)	C12—C13—C8	122.4 (2)
O1—C3—C4	115.5 (3)	C12—C13—H13A	118.8
C2—C3—C4	119.5 (2)	C8—C13—H13A	118.8
C5—C4—C3	120.2 (3)	O3—C14—H14A	109.5
C5—C4—H4A	119.9	O3—C14—H14B	109.5
C3—C4—H4A	119.9	H14A—C14—H14B	109.5
C4—C5—C6	121.8 (2)	O3—C14—H14C	109.5
C4—C5—H5A	119.1	H14A—C14—H14C	109.5
C6—C5—H5A	119.1	H14B—C14—H14C	109.5
C5—C6—C1	116.9 (2)	O1—C15—H15A	109.5
C5—C6—C7	119.8 (2)	O1—C15—H15B	109.5
C1—C6—C7	123.3 (2)	H15A—C15—H15B	109.5
O2—C7—N1	122.5 (2)	O1—C15—H15C	109.5
O2—C7—C6	121.7 (2)	H15A—C15—H15C	109.5
N1—C7—C6	115.8 (2)	H15B—C15—H15C	109.5
C13—C8—C9	115.9 (2)		
			
C6—C1—C2—C3	−1.3 (4)	C13—C8—C9—C10	0.4 (4)
C15—O1—C3—C2	−1.3 (4)	N1—C8—C9—C10	−175.8 (2)
C15—O1—C3—C4	179.6 (2)	C13—C8—C9—N2	−178.2 (2)
C1—C2—C3—O1	−178.4 (2)	N1—C8—C9—N2	5.6 (4)
C1—C2—C3—C4	0.7 (4)	O5—N2—C9—C10	17.1 (3)
O1—C3—C4—C5	−179.8 (2)	O4—N2—C9—C10	−161.4 (2)
C2—C3—C4—C5	0.9 (4)	O5—N2—C9—C8	−164.2 (2)
C3—C4—C5—C6	−2.1 (4)	O4—N2—C9—C8	17.4 (4)
C4—C5—C6—C1	1.6 (4)	C8—C9—C10—C11	−1.7 (4)
C4—C5—C6—C7	−179.6 (2)	N2—C9—C10—C11	177.0 (2)
C2—C1—C6—C5	0.2 (4)	C14—O3—C11—C12	−4.6 (4)
C2—C1—C6—C7	−178.6 (2)	C14—O3—C11—C10	175.1 (2)
C8—N1—C7—O2	−6.6 (4)	C9—C10—C11—O3	−178.6 (2)
C8—N1—C7—C6	174.1 (2)	C9—C10—C11—C12	1.1 (4)
C5—C6—C7—O2	−27.8 (4)	O3—C11—C12—C13	−179.6 (2)
C1—C6—C7—O2	150.9 (3)	C10—C11—C12—C13	0.7 (4)
C5—C6—C7—N1	151.5 (3)	C11—C12—C13—C8	−2.1 (4)
C1—C6—C7—N1	−29.7 (4)	C9—C8—C13—C12	1.5 (4)
C7—N1—C8—C13	33.7 (4)	N1—C8—C13—C12	177.7 (2)
C7—N1—C8—C9	−150.3 (3)		

### Hydrogen-bond geometry (Å, º)

**Table:**

*D*—H···*A*	*D*—H	H···*A*	*D*···*A*	*D*—H···*A*
N1—H1*A*···O2^i^	0.80 (3)	2.34 (3)	3.027 (3)	145 (3)
C10—H10*A*···O5^ii^	0.93	2.45	3.364 (3)	168

Symmetry codes: (i) *x*, *y*+1, *z*; (ii) −*x*+1, −*y*+2, −*z*.
